# Evolution of work ability, quality of life and self-rated health in a police department after remodelling shift schedule

**DOI:** 10.1186/s12889-022-14098-5

**Published:** 2022-09-03

**Authors:** Marcial Velasco-Garrido, Robert Herold, Elisabeth Rohwer, Stefanie Mache, Claudia Terschürenm, Alexandra M. Preisser, Volker Harth

**Affiliations:** Institute for Occupational and Maritime Medicine (ZfAM), University Medical Center Hamburg-Eppendorf, Hamburg, Germany

**Keywords:** Shift-work, Police, Work ability, Health, Quality of life

## Abstract

**Background:**

There exists a great diversity of schedules concerning the way shift work is organized and implemented with ample agreement regarding recommendable features of a shift system. In order to adapt the shift schedule of a metropolitan police department to current recommendations, a remodelled shift schedule was introduced in 2015. The aim of this study was to evaluate the potential associations between the remodelled shift schedule and work ability, quality of life and self-rated health after one and five years.

**Methods:**

A controlled before-and- after study was conducted during the piloting phase (2015–2016) as well as a 5-year follow-up using paper questionnaires. Outcome parameters included work ability, quality of life and self-rated health.

**Results:**

Work ability, quality of life and self-rated health improved after the first year of the newly implemented shift schedule among police officers working in the piloting police stations compared to those working according to the former schedule. In 5-year follow-up differences between indicators diminished.

**Conclusions:**

The implementation of a remodelled shift schedule including more 12-h shifts accompanied by more days off and a coherent weekend off duty was not associated with detrimental effects to work ability, quality of life or self-reported health among police officers.

**Supplementary Information:**

The online version contains supplementary material available at 10.1186/s12889-022-14098-5.

## Background

Police work is considered to be one of the most stressful occupations, with officers experiencing both physical and psycho-social stress [1]. Work-related stress is associated with lower health-related quality of life among police officers [2, 3]. Chronic psycho-social stressors in police work are usually classified as structural-organisational (related to the context of the job) and inherent to the job (related to the content of the job), also known as operational stressors [4]. Shift work, is one of the most relevant intrinsic operational psycho-social stressors affecting the health of police officers [5]. It is well known that shift work can have adverse health effects by disrupting the individual circadian rhythm [6–9]. Shift work in policing has been associated with adverse health outcomes including sleep disorders, diabetes, depression, cardiovascular risk factors and cardiovascular morbidity and mortality. [10]. In addition, shift work may lead to more conflicts with work-life balance than working hours without shift work [11].

There exists a great diversity of schedules to organize and implement shift work. Some features of shift schedules are considered to be less deleterious to health than others [12] (e.g. forward rotation vs. backward rotation). Thus, there is ample agreement regarding recommendable features of a shift system in order to reduce the risks associated with shift-work [12, 13]. The main ergonomic recommendations to organize shift-work are summarized in Table 1.

**Table 1:** Recommendations for the organisation of shift work [12, 13]

1.The number of consecutive night shifts should be as low as possible
2.A night shift phase should be followed by a recovery period as long as possible. In no case should it be less than 24 h
3.Blocked weekend breaks are better than single days off at weekends
4.Shift workers should have more days off per year than day workers
5.Unfavourable shift patterns should be avoided, i.e. always rotate forward
6.The early shift should not start too early
7.The night shift should end as early as possible
8.Rigid starting times should be avoided in favour of individual preferences
9.The concentration of working days or of working hours into one day should be limited
10.Shift schedules should be predictable and manageable

Until the year 2015, the former shift system for the uniformed police of a German major city was in discordance with some of these recommendations. In particular, there was a lack of blocked weekend breaks (i.e. allowed only single days off), which are notably important for recreation, social life and regeneration. The recovery (off duty) periods after night shifts were too short to allow for optimal regeneration, which is particularly problematic since quick returns (i.e. short rests) between shifts are detrimental to health [14]. In addition, the early shift started at 05:30 a.m, so some officers had to end their night sleep already at 03:30 a.m. in order to be on time at work. This might be associated with relevant sleep deficits depending on the individual chronotype of the officers and sleep behaviour [15]. Finally, with 40 shifts every 8 weeks, police officers had the same amount of working days as non-shift workers instead of less as recommended [13].

To address these short-comings, the shift schedule was redesigned by a working group consisting of representatives from both personnel management and staff. The remodelled shift schedule was initially introduced as a pilot project between June 2015 and June 2016 in 6 out of 24 police stations. During 2017, the schedule was adopted by the remaining police stations, with one exception that did not start implementing the shift schedule until 2021.

The two shift models are compared in Table 2. The main ergonomic benefits of the remodelled shift schedule are blocked weekends off duty, more recovery time after night duty and fewer shifts overall. However, the implementation of the remodelled shift model requires an increase in the number of 12-h shifts. Compressed work schedules with 12-h shifts are controversial. There are studies indicating higher levels of emotional and physical exhaustion and higher incidence of health complaints (headaches, musculoskeletal pain, faintness) associated with extended work shifts in comparison with 8-h shifts [16–18]. Other studies suggest however that compressed rosters with 12-h shifts are associated with higher work satisfaction, better quality of life and emotional wellbeing, better quality of sleep and less fatigue as well as improvements in work-life-balance [19–21].

**Table 2:** Characteristics of former and remodelled shift model over a rotation period of 8 weeks

	**Former shift model**	**Remodelled shift model**
Rotation	Forward	Forward
Number of shifts	40	35
Working hours	360	359
Number of 12-h shifts	4	14
Rest period (in hours) after night duty	23.75–30.75	72
Weekends off duty	0	1
Days-off^a^	2	14

^a^defined as days in which a shift neither begins nor ends

Accounting for the potential adverse effects to health of the increased number of 12-h shifts within the remodelled schedule, the metropolitan personnel office – the supervisory authority responsible for surveillance of labour legal requirements – approved the implementation of the remodelled shift schedule provided that its effects on the health and social well-being of police officers be evaluated.

This paper presents the results of the evaluation in terms of work ability and perceived health status after five years applying the remodelled shift model.

The concept of work ability captures the balance between job demands on the one side and health and functional capacity of an individual on the other side, with the Work Ability Index (WAI) being the most used instrument to measure it [22]. In addition the WAI predicts work disability and mortality [23]. For this reason we consider it an appropriate instrument to evaluate health effects in an occupational environment.

As stated above work stressors, including shift work, are associated with health, in particular with the incidence of chronic diseases. Health complaints and chronic disease have an impact on health related quality of life and on self-rated health [24]. Global self-rating of health addressed with a single question is a good predictor of morbidity and mortality [25].

The aim of our study was to investigate and answer the following research questions:

1. Is there an association between shift schedule and work ability?
2. Is there an association between shift schedule and reported quality of life?
3. Is there an association between shift schedule and self-rated health status?

## Methods

### Study design and population

We conducted a controlled before-after study during the pilot phase (2015–2016). All police stations (*n* = 24) of the department participated in the study.

The allocation to the intervention group was outside the control of the researchers. At each station polls were conducted among the affected officers. The remodelled shift schedule was implemented in the stations where more than 2/3 voted for it. The intervention group included the 6 police stations which implemented the remodelled shift schedule as of June 2015 for a period of one year. The control group included those 17 police stations which continued to operate with the current shift schedule throughout the same period of time. One station implemented the remodelled roster as of November 2015 and thus we excluded it from the controlled before-after study.

Outcome parameters were evaluated in both groups in May 2015 (1 month before starting the pilot-phase) and 12 month afterwards (June 2016).

**Fig. 1:**
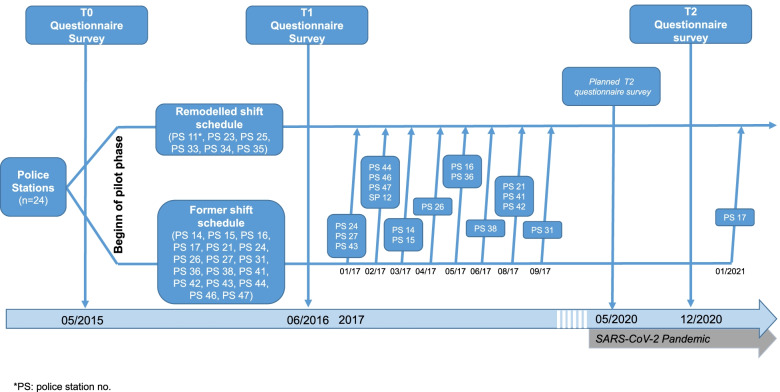
Study design

A follow-up survey was conducted in December 2020, 5.5 years after the implementation of the remodelled shift schedule. The follow-up was originally scheduled for June 2020 (i.e. 5 years after the introduction of the remodelled shift schedule) but had to be postponed due to the SARS-CoV-2 pandemic. The control group vanished between 2016 and 2020 due to the progressive adoption of the remodelled shift schedule in all police stations of the city. At this time point, the remodelled shift schedule had been already adopted by all police stations (with one exception, which implemented it as of January 2021) (see Fig. 1). The officers working in the operations command centre were also included in the third survey, since they had also adopted the remodelled shift schedule. Again, the decision to implement the remodelled shift schedule was made in each station by voting with the requirement of a majority of 2/3 of the officers. Thus, the long term follow-up corresponds to a prospective cohort study in which participants had different levels of exposure to the remodelled shift schedule – i.e., the length of time they worked with this shift schedule.

### Questionnaire

The questionnaire was paper-based and anonymous. In order to match responses over the three survey waves, participants were asked to provide a matching code consisting of a combination of letters and numbers that the participants chose themselves. The data protection officer of the Department of the Interior and the police staff council approved the content of the questionnaire and the survey method. The questionnaire was distributed among all police officers working according to the rotating shift schedule via the internal staff post. Participants had four weeks to return the filled in questionnaires. Locked and sealed ballot boxes were set up in the police stations for collecting the questionnaires. In the CBA-study one reminder was sent via email two weeks after distribution of the questionnaire, at both T0 and T1. On the third survey we did not send any reminders. In the first survey (05/2015), 1151 police officers returned valid questionnaires (72.7% response rate). Response rates of the second (06/2016) and third (12/2020) survey were similarly high (74.3% and 70.2%, respectively). After excluding non-valid questionnaires (53, 59 and 147, respectively), the de facto response rates were 69.4%, 70.4 and 61%, respectively.

### Sociodemographic variables

We collected data on gender, age (in five year categories), relationship status (‘living in a relationship’ / ‘not living in a relationship’), parenthood (‘yes’ / ‘no’), single parenting (‘yes’ / ‘no’) and taking care of dependents (‘yes’ / ‘no’).

### Job characteristics

We collected data on experience with shift rotations (‘less than 5 years’ / ‘5 to 10 year’ / ‘more than 10 years’), working full or part-time, and main type of duty (‘office duty’ / ‘patrol duty’).

### Outcome parameters

#### Work ability

Work ability was measured with the German version of the *Work Ability Index* (WAI) [26]. The WAI consists of ten questions covering the dimensions of current work ability compared to lifetime best (score 0–10), current work ability in relation to job demands (score 0–10), impairment of work performance due to illness (score 1–6), sickness leave in the past 12 months (score 1–5), anticipated work ability for the next two years (score 1–7), psychological resources (score 1–4) and number of medical conditions out of a short list of 14 [9]. The WAI score ranges from 7 to 49: Scores below 28 are referred to as ‘critical’, between 28 and 36 points as ‘moderate’, between 37 and 43 points as ‘good’, and higher scores as ‘very good’ work ability [27]. The WAI can be considered reliable (Cronbach’s α 0.78) and valid [28].

#### Self-rated health

Self-rated general health was addressed with a single question “How would you rate your health in general?” on a five-point Likert scale (‘excellent’ / ‘very good’ / ‘good’ / ‘fairly good’ / ‘poor’) as recommended by WHO [29]. For further statistical analysis we dichotomized the variable merging the categories ‘excellent’ / ‘very good’ / ‘good’ on the one side and ‘fairly good’ / ‘poor’ on the other side. In addition, we asked participants to rate their health on a 0–10 scale, where 0 represents worst imaginable health.

#### Quality of life

Quality of life was assessed with the global domain of the German version of WHOQOL-Bref [30]. It consists of two questions (“Over the last two weeks, how would you rate your quality of life?” and “Over the last two weeks, how satisfied were you with your health?”) answered on a five-point Likert scale from 1 = “very bad/unsatisfied” to 5 = “very good/satisfied. The answers are transformed into a global score ranging from 0 to 100, 100 indicating highest quality of life. The instrument in its short version can be considered reliable (Cronbach’s α ranging from 0.57 to 0.88) and valid [30]. In addition, we asked participants to rate their quality of life with the shift model on a 0–10 scale, where 0 represents worst and 10 the best imaginable quality of life.

### Statistical analysis

We did not perform any imputation for any variable, items left unanswered were treated as missing values and accordingly the corresponding scores. Descriptive statistics are reported as means with standard deviation (SD) for continuous variables, and as frequencies and percentages for categorical variables. We calculated two-tailed *p* values. The statistical significance level was set at *p* < 0.05. All computations were carried out with IBM® SPSS® Statistics (IBM Corp. released 2015. IBM SPSS Statistics for Windows, Version 25.0. Armonk, NY, USA).

#### Controlled before-after

Normally distributed score means were compared in bivariate analysis with t-test for independent samples before starting the pilot (T0) and 12 months later (T1). We calculated effect sizes for those scores showing statistically significant differences. The effect size Cohen’s d (|d|) for mean differences between two groups (comparison of mean values from the two groups) was determined as an effect measure. |d|< 0.2 is rated as insignificant, |d|≥ 0.2 to < 0.5 as small, |d|≥ 0.5 to < 0.8 as medium and |d|≥ 0.8 as large effect size [31]. For categorical variables, the chi-square test for independence was used to test for group differences in bivariate analysis.

We performed multiple linear regression according to the ordinary least squares (OLS) method with the scores of the outcome parameter at T1 as the dependent variables. The explanatory variables were the type of shift worked with (‘old schedule’ / ‘remodelled schedule’), the score values at baseline (T0), gender (‘male’ / ‘female’), age group at the time of the second survey (< 35 years, 35 – 49 years, ≥ 50 years), parenthood (‘yes’ / ‘no’), a variable representing ‘burden due to care” (‘yes’ / ‘no’), which was a composite variable of the information on status from the questions on single parenthood ‘single parent’ and ‘care of persons in need of care’, as well as the type of service (‘patrol’ / ‘office duty’). We report the coefficient with 95% confidence intervals for the predictor. For binary variables, we performed logistic regression including the same variables as in the linear regression models, with the exception of baseline score.

#### Long-term follow-up

Data from the T2 survey were first analysed in bivariate analyses stratified by the length of time working with the remodelled shift model in months (up to 24 months, 25 – 48 months, ≥ 49 months). For comparison across “exposure” categories, analyses of variance were carried out using Welch tests for correction. For this purpose, the effect size measure Eta-squared (η^2^) was used, whereby an Eta-squared of 0.01 is considered a small effect, of 0.06 a medium effect and of 0.14 a large effect [31]. We performed multivariate linear regression analyses where the dependent variable were the scores at T2. The length of time servicing with the remodelled shift schedule in months, gender (male/female), age group at the time of the third survey (≤ 34 years, 35–49 years and ≥ 50 years), having children (‘yes’ / ‘no’), care burden (‘yes / ‘no’), police station (‘originally piloting’ / ‘non-piloting’) as well as type of service (‘patrol’ / ‘office duty’) were included as explanatory variables. We determined the effect size f^2^, where an f^2^ of 0.02 corresponds to a weak effect, 0.15 represents a medium effect and 0.35 a strong effect [32]. For binary variables, we performed logistic regression including the same variables as in the linear regression models.

## Results

The characteristics of the participants are presented in Table 3. The participants’ age and gender distribution were comparable to that of the target staff at the three time points (see supplementary table 1). The participants in the third survey were younger compared to T0 and T1. Accordingly, they had less experience with shift work and familial burden. The proportion of officers working mainly on patrol duty is lower in T2 than in T0 and T1 due to the incorporation of the operations command center to the third survey.

**Table 3:** Participant ‘s sociodemographic and job characteristics (2015, 2016, 2020)

Variable	T0 (05/2015) (*n* = 1151)		T1 (06/2016) (*n* = 1122)		T2 (12/2020) (*n* = 1027)	
	**n**	**%**	**n**	**%**	**n**	**%**
Gender (female)	338	30.8	349	31.6	377	36.9
Age distribution						
20–24 yrs	31	2.7	30	2.7	110	10.8
25–29 yrs	121	10.6	142	12.7	195	19.1
30–34 yrs	234	20.5	196	17.5	173	16.9
35–39 yrs	226	19.8	238	21.2	151	14.8
40–44 yrs	149	13.0	149	13.3	159	15.6
45–49 yrs	176	15.4	151	13.5	79	7.7
50–54 yrs	173	15.1	162	14.4	75	7.3
≥ 55 yrs	34	3.0	45	4.0	80	7.8
Age (weighted mean. CI in yrs.)	39.6	37.5; 41.7	39.5	37.4; 41.6	37.1	34.9; 39.2
Parenthood (yes)	644	57.9	614	57.3	464	46.1
Burden due to care (yes)	79	7.9	64	6.6	67	7.5
Experience with shift rotations (yrs.)						
< 5 yrs	167	14.6	198	17.8	344	33.7
5–10 yrs	196	17.1	152	13.7	219	21.4
> 10 yrs	784	68.1	763	68.6	459	44.9
Mainly patrol duty	824	71.8	791	71.1	685	66.8
Fulltime job	1005	87.6	957	85.3	901	88.2

Table 4 shows the overall results for the outcome parameters at the three survey time points. Overall, work ability, quality of life, and health status were rated lower before the remodelled shift schedule was implemented and highest five years thereafter.

**Table 4:** Outcome parameters (2015, 2016, 2020)

Outcome	T0 (05/2015)		T1 (06/2016)		T2 (12/2020)	
	**mean**	**SD**	**mean**	**SD**	**mean**	**SD**
Work Ability Index [Score: 7–49]	38.11	5.72	39.06	5.55	40.34	5.01
WHOQOL-Bref Global [Score. 0–100]	59.84	19.94	64.43	19.45	67.93	18.18
Quality of life with shift schedule [Score: 0–10]	5.21	1.97	5.72	2.09	6.71	1.60
Self-rated health status score [Score: 0–10]	6.65	1.90	6.95	1.77	7.30	1.58
Self-rated health status	**n**	**%**	**n**	**%**	**n**	**%**
excellent	39	3.4	52	4.7	84	8.2
very good	307	26.8	313	28.0	378	37.0
good	600	52.4	609	54.5	475	46.5
fairly good	183	16.0	135	12.1	79	7.7
poor	15	1.3	8	0.7	5	0.5

### Controlled before and after pre-post analysis

For the controlled before and after analysis (T0-T1) a total of 583 valid questionnaires could be matched. The characteristics of this subgroup were similar to the characteristics of the total sample in T0 and T1 (see supplementary table 2).

At baseline, work ability, quality of life and health status were comparable between the intervention and the control group (see Table 5). After one year, work ability improved slightly in the group piloting the remodelled shift schedule, while it tended to deteriorate in the group with the former shift model. The same was observed for the health status score. Quality of life improved in both groups, but the increase was stronger in the group with the remodelled shift model. All differences were statistically significant. The effect sizes ranged from small to strong.

**Table 5:** Controlled before and after (2015, 2016)

	**T0 (05/2015)**					**T1 (06/2016)**					**Change (T1-T0)**					
	**former shift**		**remodelled shift**			**former shift**		**remodelled shift**			**former shift**		**remodelled shift**			**Effect size** **|d|**
	**mean**	**SD**	**mean**	**SD**	**p**	**mean**	**SD**	**mean**	**SD**	**p**	**mean**	**SD**	**mean**	**SD**	**p**	
Work Ability Index (*n* = 360) [Score: 7–49]	39.05	5.45	39.19	5.28	0.828	38.93	5.72	40.41	4.94	0.023	-0.12	4.97	1.22	4.42	0.019	0.28
WHOQOL-Bref Global (*n* = 578) [Score: 0–100]	61.74	18.99	62.04	19.77	0.869	62.59	20.07	71.42	16.66	< 0.01	0.84	19.57	9.37	22.81	< 0.001	0.40
Quality of life with shift schedule (*n* = 567) [Score: 0–10]	5.42	1.93	5.27	1.89	0.425	5.43	1.99	6.88	1.71	< 0.01	0.01	1.65	1.61	2.72	< 0.001	0.80
Self-rated health status score (*n* = 576) [Score: 0–10]	6.87	1.74	6.87	1.92	0.986	6.80	1.78	7.42	1.52	< 0.01	-0.08	1.59	0.55	1.94	< 0.001	0.77

**Fig. 2:**
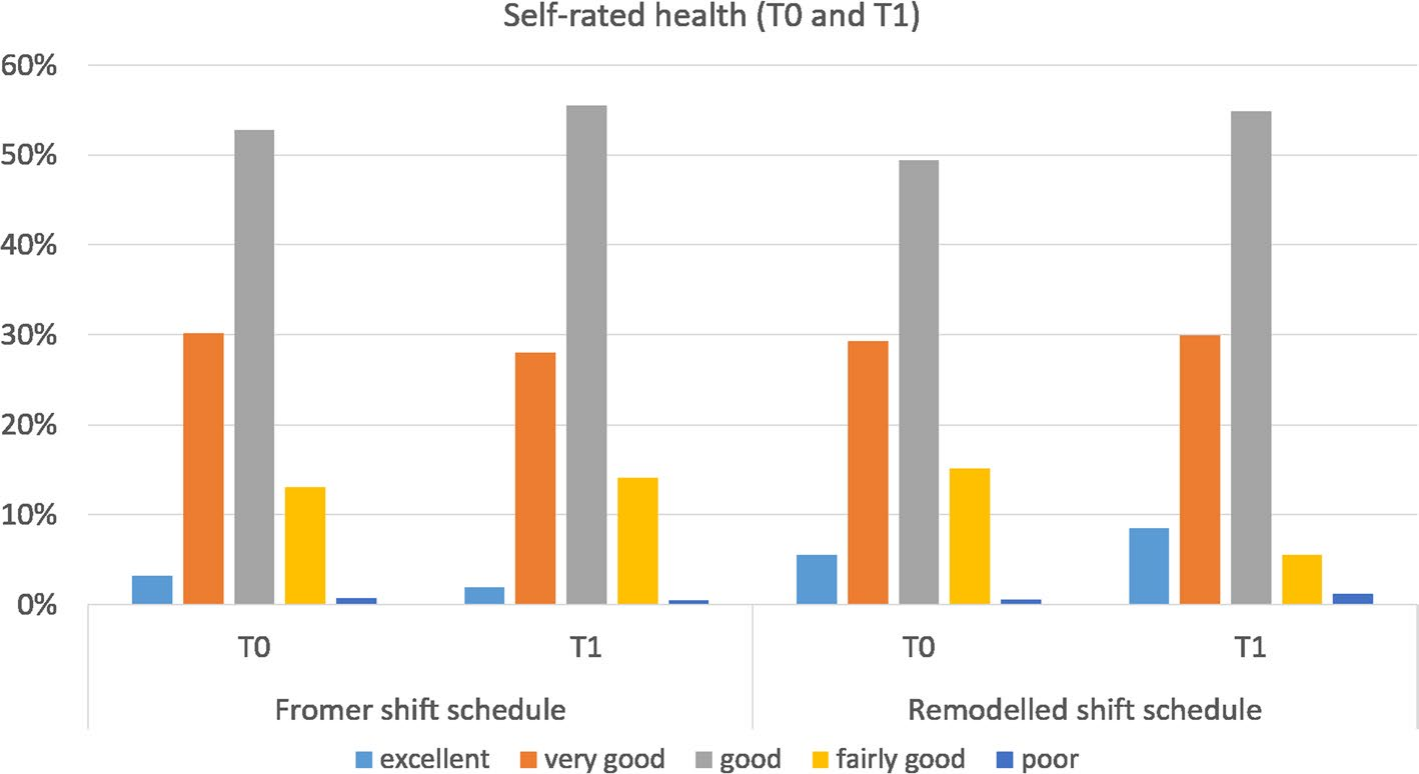
Distribution of answers to self-rated-health question (T0 and T1)

Figure 2 depicts the results of the assessment of health status on the Likert-scale. At baseline, there was no statistically significant difference in the distribution of answers, with 86.2% of police officers in the former shift schedule group and 84.1% in the remodelled shift schedule group reporting “good” to “excellent” health. After one year, these answers summed up to 93.3% in the remodelled shift group while stayed at 85.3% in the control group. The matched analysis showed a statistically significant higher risk of reporting poor health after one year among the police officers in the control group (RR: 2.19, 95% CI: 1.18 – 4.05). The difference was statistically significant in the multivariate logistic regression model (OR: 2.33, 95% CI: 1.18 – 4.59, *p* = 0.014). Multicollinearity was not relevant in the logistic regression model (variance inflation factor (VIF): 1.341) although the model had low explanatory power (R^2^ = 0.049).

Table 6 shows the results of the multivariate analysis for the continuous outcome parameters. The data satisfied the assumptions for the OLS-regression and no relevant multicollinearity was found in any of the models (the VIF ranged between 1.014 and 2.086) (see supplementary Tables 3 to 6). All models showed a statistical significant association between working with the remodelled shift schedule and higher scores for work ability, quality of life and health status. The effect sizes are strong, with the exception of the WHOQOL-Bref-Global Score, for which the effect size is medium.

**Table 6:** Multivariate analysis, effect of remodelled shift (2015, 2016)

	**n**	**Coefficient**	**95% CI**		**p**	**R**^**2**^	**Adjusted R**^**2**^	**p for model**	**Effect size f**^**2**^
			**LL**	**UL**					
Work Ability Index [Score: 7–49]	350	1.231	0.184	2.278	0.021	0.398	0.383	< 0.0001	0.661
WHOQOL-Bref Global [Score: 0–100]	557	8.365	5.121	11.609	< 0.0001	0.224	0.213	< 0.0001	0.289
Quality of life with shift model [Score: 0–10]	448	1.479	1.153	1.805	< 0.0001	0.286	0.294	< 0.0001	0.400
Self-rated health status score [Score: 0–10]	556	0.602	0.331	0.874	< 0.0001	0.304	0.276	< 0.0001	0.437

Predictors: Score at baseline T0, gender, age group, parenthood, burden of care, patrol duty

After one year, police officers working with the remodelled shift schedule showed a higher WAI, in average 1.231 points (95% CI: 0.184 – 2.278, *p* = 0.021). Being female was also a relevant predictor of WAI after one year, although associated with lower scores (-1.486, 95% CI: -0.363 – -2.609, *p* = 0.010; see supplementary Table 3).

Similarly WHOQOL-Bref showed statistically significant higher scores in the intervention group (8.365, 95%CI: 5.121 – 11.609, *p* < 0.0001). In this case, age over 50 years was associated with higher quality of life (5.810 95% CI: 0.775 – 10.845, *p* = 0.024; see supplementary Table 4). A strong effect was also observed in the 0–10 quality of life scale with 1.479 higher scores in the intervention group (95% CI: 1.153 – 1.805, *p* < 0.0001).

Finally self-rated health status was on average 0.602 points higher in the intervention group after one year (95% CI: 0.331 – 0.874, *p* < 0.0001).

### Long-term follow-up

Table 7 shows the outcome parameters stratified by the length of period the police officers worked according to the remodelled shift schedule. All scores decreased with increasing periods working with the remodelled shift schedule, the differences being statistically significant for all scores but the work ability index. The effect sizes were small for all scores. In the models adjusting for age, gender, familial burden and type of duty, the data satisfied the assumptions for the OLS-regression and no relevant multicollinearity was found in any of the models (the VIF ranged between 1.028 and 1.977) (see supplementary Tables 7 to 10). The coefficients indicate a minimal decrease in the respective scores with increasing periods working with the remodelled schedule, although none of the differences were statistically significant (see Table 8).

**Table 7:** Long-term analysis. Results stratified by length of work with remodelled shift schedule

	**Outcome**											
	**Work Ability Index [Score 7–49]**			**WHOQOL-Bref Global [Score 0–100]**			**Quality of life with shift schedule [Score 0–10]**			**Self-rated health status score [Score 0–10]**		
**Working with the remodelled shift schedule…**	**mean**	**SD**	**n**	**mean**	**SD**	**n**	**mean**	**SD**	**n**	**mean**	**SD**	**n**
** ≤ 24 months**	40.91	4.85	232	71.34	17.14	239	7.04	1.41	240	7.59	1.47	239
**25–48 months**	40.14	5.20	400	68.11	18.09	408	6.66	1.59	409	7.32	1.53	407
** ≥ 49 months**	40.39	4.83	256	65.74	18.54	262	6.63	1.72	263	7.14	1.68	262
**p**	0.171			0.002			0.002			0.006		
**Effect size η**^**2**^	-			0.013			0.01			0.01

**Table 8:** Multivariate analysis, effect of length of work with remodelled shift

	**n**	**Coefficient**	**95% CI**		**p**	**R**^**2**^	**Adjusted R**^**2**^	**p for model**	**Effect size f**^**2**a^
			**LL**	**UL**					
Work Ability Index [Score 7–49]	759	-0.005	-0.028	0.018	0.650	0.005	-0.005	0.848	-
WHOQOL-Bref Global [Score 0–100]	768	-0.065	-0.144	0.025	0.112	0.020	0.010	0.044	-
Quality of life with shift model [Score 0–10]	779	-0.003	-0.010	0.004	0.398	0.033	0.023	0.001	-
Self-rated health status score [Score 0–10]	776	0.000	-0.007	0.006	0.902	0.019	0.009	0.061	-

Predictors: gender. age group, parenthood, burden of care, working in an originally piloting police station, patrol duty

^a^No effect size was calculated, since none of the coefficients were statistically significant

The work ability decreased 0.005 points with each month working with the remodelled shift schedule (95% CI: -0.028 – 0.018, *p* = 0.650), the self-rated health status did not change at all (0.000, 95% CI -0.007 – 0.006, *p* = 0.902).

Although statistically not significant, the decrease in quality of life measured with WHOQOL-Bref was stronger (0.065 point for each month, 95% CI: -0.144 – 0.015, *p* = 0.112) as the one measured with the 0–10 scale the decrease (-0.003, 95% CI: -0.010 – 0.004) according to the standardized coefficients (-0.063 vs. -0.033). In both cases working in one of the stations which originally piloted the remodelled schedule was associated with higher quality of life (WHOQOL-Bref: 3.689. 95% CI: 0.729 – 6.650, *p* = 0.015; Score(_0–10)_: 0.309. 95% CI: 0.046 – 0.572, *p* = 0.021; see supplementary Tables 8 and 9).

**Fig. 3:**
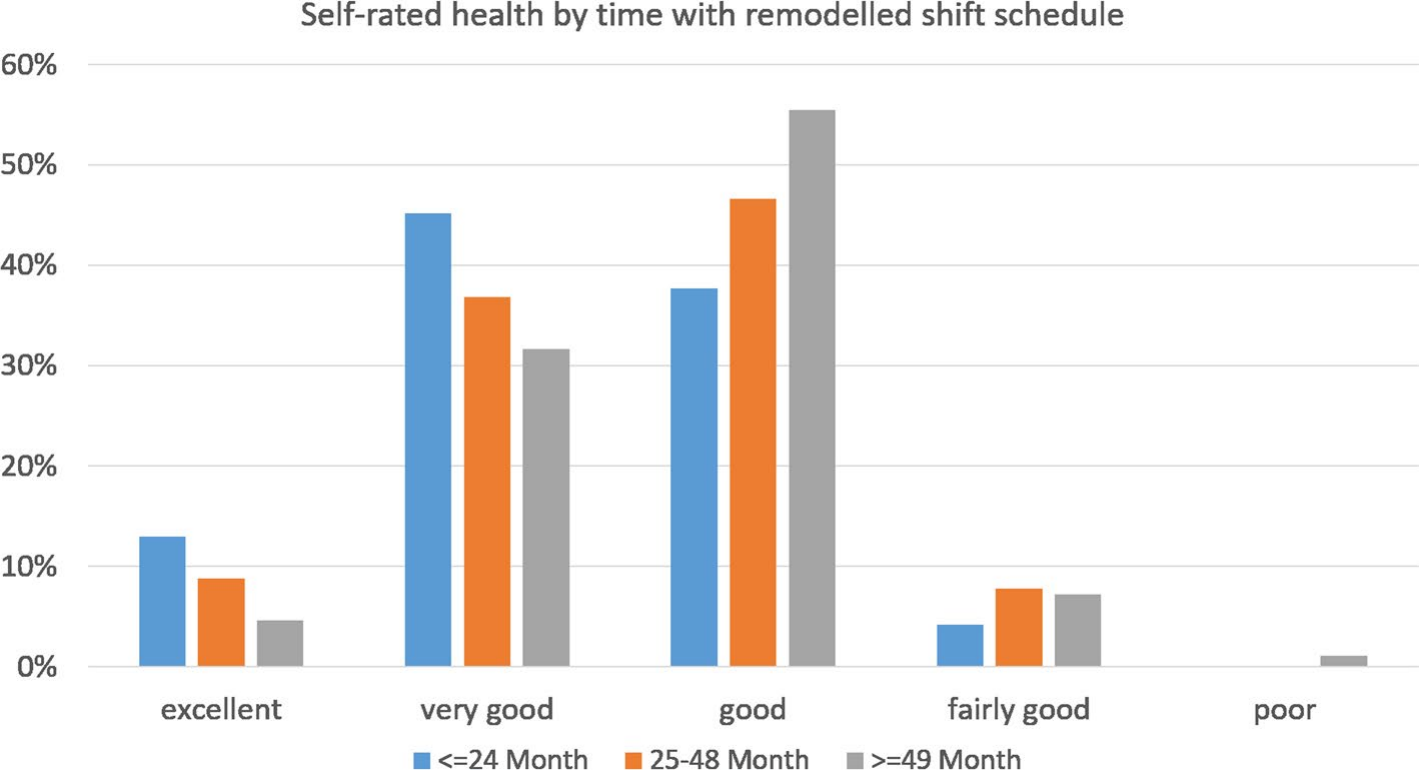
Distribution of answers to self-rated health question by length of work with the remodelled shift schedule

Figure 3 shows the answers to the question regarding the assessment of health status on the Likert-scale distributed among the three categories of exposure to the remodelled shift schedule. With increasing exposure, the proportion of officers reporting their health as “good” to “excellent” decreased from 95.9% to 91.7%. The difference in the distribution of answers is statistically significant (*p* < 0.0001). In the multivariate analysis, the association between length of period working with the remodelled shift schedule and risk of reporting poor health was not statistically significant (OR: 1.00, 95% CI: 0.98 – 1.01, *p* = 0.625). None of the predictors included in the model showed a statistically significant association with health status. Multicollinearity was not relevant in the logistic regression model (VIF: 1.433) but the model had low explanatory power (R^2^ = 0.033).

## Discussion

The stepwise introduction of a remodelled shift model allowed to design a comparative evaluation to assess the potential effects on work ability, quality of life and self-reported health among police officers. Our results indicate that the remodelled shift schedule may have had positive effects on these outcome parameters in the first year. The follow-up after five years suggests that the initial positive effect slowly disappears as the time working with this schedule increases.

Since the evaluation was a prerequisite for the piloting of the remodelled shift schedule and a basis for decision-making regarding its subsequent implementation, police officers’ attitude towards the remodelled shift schedule might have affected their motivation to participate in the survey and may have also biased their answers. Different response rates at the police department level may indicate this as well as the results of comparing piloting versus non-piloting police departments in the controlled before-after analysis. Indeed, the proportion of police officers willing to change the shift system was 81.3% in the group piloting the remodelled shift, compared to 57.6% in the group not piloting. The baseline scores of the officers willing to change to the remodelled shift schedule were significantly lower at baseline and increased more in the first year than those not willing to change the schedule (see supplementary Table 11). Initial positive effects after the awaited piloting may have faded away over time in the daily work routine.

Although we cannot draw conclusions on separate aspects of the shift schedule, it is plausible that the large number of over-length shifts (12-h shifts) within the schedule may have a relevant impact in the vanishing of the initial positive effects over time. Compressed work schedules with rotas of 12-h or longer have been associated with higher levels of fatigue among police officers [33, 34]. Long working hours has been identified as a one of the organisational stressors that may increase the risk of emotional exhaustion and psychological distress among law enforcement officers [35]. Recently a survey among 3410 US-police officers working with different shift schedules has shown a dose–response association between the number of long-shifts (≥ 11 h) per week and a high degree (≥ 27 points) of emotional exhaustion dimension of the Maslach Burnout Inventory with higher risk already with one long-shift per week [36]. The schedule studied by us includes two long-shifts per week. Indeed, the number of 12-h shifts was the most frequently mentioned disadvantage of the evaluated shift schedule by the participants in our survey [37]. These long shifts had been described previously by the police officers in our study in qualitative interviews as being highly demanding, particularly the 12-h overnight shifts (from 06:00 p.m. to 06:00 a.m.) [38]. However, the multivariate analysis adjusting for age (among other factors) allowed us to conclude that there is no statistically significant association between long-term work with the remodelled shift schedule and detrimental effects to self-reported health or quality of life. This might be an effect of the more frequent and longer recovery periods within this schedule. Findings from the industry indicate that schedules preventing shift worker from summing up chronic sleep deficits may reduce adverse effects of shift work irrespective of shift duration [39].

Overall, the police officers in our study showed a good work ability [40], comparable to that of a nationwide representative sample of employees aged 31–60 years in Germany (M = 40.22; SD 6.20) [41]. The work ability index improved in the first year among those working with the remodelled schedule. In the long term, we did not observe any relevant decrease in the work ability index – taking into account the effects of age – in relation to working longer with the remodelled schedule, although it is well known that the WAI has a tendency to decline with age [42]. At the individual level, the WAI is able to identify workers at high risk of long-term sickness absence [43], thus good work ability – as seen in our samples – can be considered to be a good indicator of general health. Recent research on the factorial validity of the WAI among employees in Germany suggests that there are two correlated factors underlying the WAI [41]. For subsequent analyses, it can provide more insight to examine the "subjective work ability and resources" and "health related" factors of the WAI separately.

We further addressed self-rated health with a categorical question, as recommended by WHO and additionally with an ad hoc 0–10 scale. Overall self-rated health has been showed to be a strong predictor of mortality, independent of the instrument used [26]. Whereas the short-term evaluation after one year shows a higher risk of poor health among the officers working with the old shift schedule, the associations again disappears over time. The long-term analysis showed no difference in self-rated health neither with the categorical question nor with the scale rating. This results are in accordance with the analysis of routine sickness leave data of the police department, which show a positive development of this parameter over time [Herold et al., in preparation].

Compared to the available WHOQOL-Bref norm values for the whole population in Germany (67.59 SD 17.93) [30], quality of life in our sample was rated lower at the baseline (M = 59.84; SD 19.94) but was comparable in the follow-up survey (M = 67.93; SD 18.18). Considering that the norm values include age groups over 65 years which may have lower levels of quality of life, our results indicate lower levels of quality of life among police officers. A cross-sectional study among criminal police officers recently showed lower health-related quality of life scores compared to results from the general population [44]. A slight decrease in quality of life, as measured with the ad hoc 0–10 scale, was also evident in our analysis in association with longer work with the remodelled shift schedule, which, however, was not statistically significant.

Due to the incremental implementation of the remodelled shift schedule, the data can only reflect effects from a maximum of five and a half years of experience with this shift schedule (Table 9). Since the observed positive effects on the short term vanished over time, the question for future research is whether working with the remodelled shift schedule over more than 5 years leads to detrimental effects on health and quality of life.

**Table 9:** What this study adds

● The incremental implementation of a remodelled shift-schedule in a police department facilitates evaluation of health related outcomes
● A shift-schedule fulfilling ergonomic recommendations is likely to improve health and wellbeing of police officers on the short-term
● A shift schedule with recurrent 12-h rotas and more work-free days does not appear to be detrimental to overall health

### Strengths and limitations

As in other studies on occupational health, one main limitation of our study is that we cannot rule out a healthy-worker effect [45]. The questionnaire was distributed in the police stations among active officers. It is possible that during the 5 year period some officers have left their work due to health problems, which would lead to an underestimation of detrimental effects of the shift schedule. Unfortunately we did not have access to data regarding to illness-related retirements. In general, the staff of the police department has changed over time due to scheduled retirements and recruitment of new staff. The average age now is 3.4 years younger than 2015, when the first survey was conducted. In addition, there has been an increase in the proportion of female police officers. These demographic changes explain the differences reported in Table 3, when comparing the cross-sectional results of the three surveys. We have accounted for these confounders in the multivariate analysis.

Although the study samples at the three time points were representative for the whole target staff regarding age and gender distribution, the participation differed considerably among police stations. There were stations with participation rates under 50% and others with response rates over 70%. In addition, in the third survey we had to exclude 9% of the questionnaires, since the participants did not tick the box providing participation consent.

Finally, the small coefficients of determination (R-squared) calculated for all our multivariate models (see Tables 6 and Table 8) indicate that particularly on the long-term factors relevantly affecting the outcome parameters could not be captured with our data.

## Conclusion

In conclusion, the change of the shift schedule including more 12-h shifts, more free days and even a free weekend was not associated with detrimental effects to work ability, quality of life or self-reported health of the police officers. However, the positive effects observed one year after implementation faded out over time which could be effected by the increased number of 12-h shifts. This issue should be addressed in the regular individual health check-ups of the officers. Future research should address the effects of high number of 12-h over more than 5 years.

## Supplementary Information

**Additional file 1:** **Supplementary Table 1.** Representativity regarding female quote and age. **Supplementary Table 2.** Participants’ sociodemographic and job characteristics (2015, 2016 and matched subgroup). **Supplementary Table 3.** Controlled before and after pre-post analysis – Multivariate analysis Work Ability Index. **Supplementary Table 4.** Controlled before and after pre-post analysis – Multivariate analysis WHOQOL-Bref Global. **Supplementary Table 5.** Controlled before and after pre-post analysis – Multivariate analysis Quality of life with shift model (Score 0-10). **Supplementary Table 6.** Controlled before and after pre-post analysis – Multivariate analysis Self-rated health status (Score 0-10). **Supplementary Table 7.** Long-term follow-up – Multivariate analysis Work Ability Index. **Supplementary Table 8.** Long-term follow-up – Multivariate analysis WHOQOL-Bref Global. **Supplementary Table 9.** Long-term follow-up – Multivariate analysis Quality of life with shift model (Score 0-10). **Supplementary Table 10.** Long-term follow-up – Multivariate analysis Self-rated health status (Score 0-10). **Supplementary Table 11.** Scores according to attitude regarding change to the remodelled shift schedule.

## Data Availability

The data cannot be made available due to contractual provisions with the sponsor regarding safety and data protection. If someone wants to request the data from this study contact Dr. Marcial Velasco Garrido (m.velasco-garrido@uke.de).
