# Supplementary material for: Evolution of work ability, quality of life and self-rated health in a police department after remodelling shift schedule
Source: BMC Public Health. 2022 Sep 3;22:1670. doi: 10.1186/s12889-022-14098-5 (PMC9439718; doi:10.1186/s12889-022-14098-5)
Supplement: Supplementary file 1 — Additional file 1: Supplementary Table 1. Representativity regarding female quote and age. Supplementary Table 2. Participants’ sociodemographic and job characteristics (2015, 2016 and matched subgroup). Supplementary Table 3. Controlled before and after pre-post analysis – Multivariate analysis Work Ability Index. Supplementary Table 4. Controlled before and after pre-post analysis – Multivariate analysis WHOQOL-Bref Global. Supplementary Table 5. Controlled before and after pre-post analysis – Multivariate analysis Quality of life with shift model (Score 0-10). Supplementary Table 6. Controlled before and after pre-post analysis – Multivariate analysis Self-rated health status (Score 0-10). Supplementary Table 7. Long-term follow-up – Multivariate analysis Work Ability Index. Supplementary Table 8. Long-term follow-up – Multivariate analysis WHOQOL-Bref Global. Supplementary Table 9. Long-term follow-up – Multivariate analysis Quality of life with shift model (Score 0-10). Supplementary Table 10. Long-term follow-up – Multivariate analysis Self-rated health status (Score 0-10). Supplementary Table 11. Scores according to attitude regarding change to the remodelled shift schedule. [file 12889_2022_14098_MOESM1_ESM.docx]

**Additional file 1. Supplementary Tables.**

**Supplementary Table 1. Representativity regarding female quote and age**

|  | **Total** | **Female** | | | **Age [yrs.]** | | |
| --- | --- | --- | --- | --- | --- | --- | --- |
|  |  | **[n]** | **[%]** | **[p]^b^** | **Average** | **LL** | **UL** |
|  | **N** |  |  |  |  |  |  |
| ***First Survey (T0)*** |  |  |  |  |  |  |  |
| Police officers (June 2015)^a^ | 1583 | 513 | 32.4 | - | 40.7^c^ | - | - |
| Valid questionnaires T0 | 1098 | 338 | 30.8 | 0.250 | 39.6^d^ | 37.5 | 41.7 |
| ***Second Survey (T1)*** |  |  |  |  |  |  |  |
| Police officers (June 2016) | 1511 | 471 | 31.2 | - | 40.5^c^ |  |  |
| Valid questionnaires T1 | 1063 | 332 | 31.2 | 0.966 |  |  |  |
| Matched sample (T0-T1) | 583 | 186 | 31.9 | 0.703 | 40.8^d^ | 38.6 | 42.9 |
| ***Third Survey (T2)*** |  |  |  |  |  |  |  |
| Police officers (December 2020) | 1673 | 583 | 34.8 | - | 37.83^c^ | - | - |
| Valid questionnaires T2 | 1023 | 377 | 36.9 | 0.311 | 37.08^d^ | 34.9 | 39.2 |
| ^a^ Except PS 37; ^b^ Chi²-Test; ^c^ Mean, provided by human resources department of the police no upper or lower limits available; ^d^ weighted mean; LL: lower limit; UL: upper limit | | | | | | | |

**Supplementary Table 2. Participants’ sociodemographic and job characteristics (2015, 2016 and matched subgroup).**

| **Variable** | **T0 (05/2015)**  **(n=1151)** | | **T1 (06/2016)**  **(n=1122)** | | **Matched T0-T1**  **(n=583)** | |
| --- | --- | --- | --- | --- | --- | --- |
|  | **n** | **%** | **n** | **%** | **n** | **%** |
| Gender (female) | 338 | 30.8 | 349 | 31.6 | 186 | 32.2 |
| Age distribution |  |  |  |  |  |  |
| 20-24 yrs. | 31 | 2.7 | 30 | 2.7 | 10 | 1.7 |
| 25.29 yrs. | 121 | 10.6 | 142 | 12.7 | 57 | 9.8 |
| 30-34 yrs. | 234 | 20.5 | 196 | 17.5 | 95 | 16.3 |
| 35-39 yrs. | 226 | 19.8 | 238 | 21.2 | 121 | 20.8 |
| 40-44 yrs. | 149 | 13.0 | 149 | 13.3 | 83 | 14.2 |
| 45-49 yrs. | 176 | 15.4 | 151 | 13.5 | 89 | 15.3 |
| 50-54 yrs. | 173 | 15.1 | 162 | 14.4 | 97 | 16.6 |
| ≥55 yrs. | 34 | 3.0 | 45 | 4.0 | 27 | 4.6 |
| Age (weighted mean. CI in yrs.) | 39.6 | 37.5; 41.7 | 39.5 | 37.4; 41.6 | 40.8 | 38.6; 42.9 |
| Parenthood (yes) | 644 | 57.9 | 614 | 57.3 | 371 | 64.0 |
| Burden due to care (yes) | 79 | 7.9 | 64 | 6.6 | 44 | 7.7 |
| Experience with shift rotations (yrs.) |  |  |  |  |  |  |
| <5 yrs. | 167 | 14.6 | 198 | 17.8 | 61 | 10.5 |
| 5-10 yrs. | 196 | 17.1 | 152 | 13.7 | 82 | 14.1 |
| >10 yrs. | 784 | 68.1 | 763 | 68.6 | 440 | 75.5 |
| Mainly patrol duty | 824 | 71.8 | 791 | 71.1 | 381 | 65.4 |
| Full-time job | 1005 | 87.6 | 957 | 85.3 | 503 | 86.3 |

**Supplementary Table 3. Controlled before and after pre-post analysis – Multivariate analysis Work Ability Index.**

|  | **Reference** | **Coefficients** | | **95% CI for B** | | **p** | **Collinearity** | |
| --- | --- | --- | --- | --- | --- | --- | --- | --- |
|  |  | **B** | **Beta** | **LL** | **UL** |  | **Tolerance** | **VIF** |
| Constant |  | 17.008 |  | 13.028 | 20.989 | <0.001 |  |  |
| Remodelled shift | old shift | 1.231 | 0.098 | 0.184 | 2.278 | 0.021 | 0.979 | 1.021 |
| WAI at T0 | (cont.) | 0.617 | 0.601 | 0.531 | 0.703 | <0.001 | 0.971 | 1.030 |
| Gender | male | -1.486 | -0.120 | -2.609 | -0.363 | 0.010 | 0.830 | 1.205 |
| Age 35 -49 yrs. | age <35 yrs. | -0.738 | -0.066 | -1.891 | 0.415 | 0.209 | 0.642 | 1.559 |
| Age >50 yrs. | age <35 yrs. | 0.129 | 0.009 | -1.465 | 1.724 | 0.873 | 0.522 | 1.915 |
| Children | none | 0.170 | 0.015 | -0.911 | 1.250 | 0.758 | 0.771 | 1.297 |
| Burden of care | none | 0.652 | 0.028 | -1.396 | 2.699 | 0.532 | 0.899 | 1.112 |
| Patrol duty | office duty | -0.201 | -0.017 | -1.319 | 0.917 | 0.724 | 0.760 | 1.316 |
| *Modell parameters* n=350, R²= 0.398, adjusted R²= 0.383, p<0,0001, average VIF= 1.307 | | | | | | | | |
| B: non-standardized coefficient; Beta: standardized coefficient; LL: lower limit; UL: upper limit; VIF: variance inflation factor. | | | | | | | | |

**Supplementary Table 4. Controlled before and after pre-post analysis – Multivariate analysis WHOQOL-Bref Global.**

|  | **Reference** | **Coefficients** | | **95% CI for B** | | **p** | **Collinearity** | |
| --- | --- | --- | --- | --- | --- | --- | --- | --- |
|  |  | **B** | **Beta** | **LL** | **UL** |  | **Tolerance** | **VIF** |
| Constant |  | 30.770 |  | 23.020 | 38.521 | <0.001 |  |  |
| Remodelled shift | old shift | 8.365 | 0.192 | 5.121 | 11.609 | <0.001 | 0.987 | 1.014 |
| WHOQOL at T0 | (cont.) | 0.439 | 0.428 | 0.362 | 0.515 | <0.001 | 0.984 | 1.016 |
| Gender | male | 1.826 | 0.043 | -1.522 | 5.173 | 0.285 | 0.859 | 1.164 |
| Age 35 -49 yrs. | age <35 yrs. | 2.498 | 0.064 | -1.206 | 6.202 | 0.186 | 0.610 | 1.640 |
| Age >50 yrs. | age <35 yrs. | 5.810 | 0.123 | 0.775 | 10.845 | 0.024 | 0.483 | 2,071 |
| Children | none | -0.268 | -0.007 | -3.571 | 3.034 | 0.873 | 0.823 | 1.215 |
| Burden of care | none | 0.864 | 0.011 | -4.903 | 6.630 | 0.769 | 0.945 | 1.058 |
| Patrol duty | office duty | -0.352 | -0.009 | -3.764 | 3.060 | 0.840 | 0.790 | 1.266 |
| *Modell parameters* n= 557, R²= 0.224, adjusted R²= 0.213, p<0,0001, average VIF= 1.305 | | | | | | | | |
| B: non-standardized coefficient; Beta: standardized coefficient; LL: lower limit; UL: upper limit; VIF: variance inflation factor. | | | | | | | | |

**Supplementary Table 5. Controlled before and after pre-post analysis – Multivariate analysis Quality of life with shift model (Score 0-10)**

|  | **Reference** | **Coefficients** | | **95% CI for B** | | **p** | **Collinearity** | |
| --- | --- | --- | --- | --- | --- | --- | --- | --- |
|  |  | **B** | **Beta** | **LL** | **UL** |  | **Tolerance** | **VIF** |
| Constant |  | 2.898 |  | 2.211 | 3.585 | <0.001 |  |  |
| Remodelled shift | old shift | 1.479 | 0.327 | 1.153 | 1.805 | <0.001 | 0.985 | 1.015 |
| QoL_(0-10)_ at T0 | (cont.) | 0.443 | 0.419 | 0.367 | 0.520 | <0.001 | 0.975 | 1.026 |
| Gender | male | 0.030 | 0.007 | -0.307 | 0.368 | 0.860 | 0.843 | 1.186 |
| Age 35 -49 yrs. | age <35 yrs. | -0.277 | -0.068 | -0.646 | 0.092 | 0.141 | 0.614 | 1.628 |
| Age >50 yrs. | age <35 yrs. | -0.100 | -0.020 | -0.599 | 0.400 | 0.696 | 0.491 | 2.036 |
| Children | none | 0.329 | 0.078 | -0.002 | 0.660 | 0.051 | 0.819 | 1.220 |
| Burden of care | none | 0.153 | 0.020 | -0.410 | 0.715 | 0.594 | 0.937 | 1.068 |
| Patrol duty | office duty | 0.065 | 0.015 | -0.275 | 0.404 | 0.709 | 0.798 | 1.254 |
| *Modell parameters* n= 448, R²= 0.286. adjusted R²= 0.294. p<0.0001. average VIF= 1.304 | | | | | | | | |
| B: non-standardized coefficient; Beta: standardized coefficient; LL: lower limit; UL: upper limit; VIF: variance inflation factor. | | | | | | | | |

**Supplementary Table 6. Controlled before and after pre-post analysis – Multivariate analysis Self-rated health status (Score 0-10)**

|  | **Reference** | **Coefficients** | | **95% CI for B** | | **p** | **Collinearity** | |
| --- | --- | --- | --- | --- | --- | --- | --- | --- |
|  |  | **B** | **Beta** | **LL** | **UL** |  | **Tolerance** | **VIF** |
| Constant |  | 3.169 |  | 2.458 | 3.881 | <0.001 |  |  |
| Remodelled shift | old shift | 0.602 | 0.156 | 0.331 | 0.874 | <0.001 | 0.986 | 1.014 |
| Health_(0-10)_ at T0 | (cont.) | 0.509 | 0.527 | 0.441 | 0.578 | <0.001 | 0.972 | 1.029 |
| Gender | male | 0.024 | 0.006 | -0.257 | 0.304 | 0.868 | 0.859 | 1.164 |
| Age 35 -49 yrs. | age <35 yrs. | -0.009 | -0.003 | -0.320 | 0.302 | 0.955 | 0.606 | 1.651 |
| Age >50 yrs. | age <35 yrs. | -0.089 | -0.021 | -0.513 | 0.335 | 0.681 | 0.479 | 2.086 |
| Children | none | 0.158 | 0.044 | -0.119 | 0.435 | 0.264 | 0.822 | 1.216 |
| Burden of care | none | -0.032 | -0.005 | -0.516 | 0.451 | 0.895 | 0.939 | 1.065 |
| Patrol duty | office duty | 0.043 | 0.012 | -0.243 | 0.329 | 0.768 | 0.789 | 1.268 |
| *Modell parameters* n= 556, R²= 0.304. adjusted R²= 0.294. p<0.0001. average VIF= 1.311 | | | | | | | | |
| B: non-standardized coefficient; Beta: standardized coefficient; LL: lower limit; UL: upper limit; VIF: variance inflation factor. | | | | | | | | |

**Supplementary Table 7. Long-term follow-up – Multivariate analysis Work Ability Index.**

|  | **Reference** | **Coefficients** | | **95% CI for B** | | **p** | **Collinearity** | |
| --- | --- | --- | --- | --- | --- | --- | --- | --- |
|  |  | **B** | **Beta** | **LL** | **UL** |  | **Tolerance** | **VIF** |
| Constant |  | 40.694 |  | 38.954 | 42.433 | <0.001 |  |  |
| Length of work with remodelled shift (months) | (cont.) | -0.005 | -0.018 | -0.028 | 0.018 | 0.650 | 0.804 | 1.244 |
| Gender | male | -0.125 | -0.012 | -0.924 | 0.674 | 0.310 | 0.919 | 1.088 |
| Age 35 -49 yrs. | Age <35 yrs. | -0.671 | -0.064 | -1.722 | 0.379 | 0.417 | 0.509 | 1.966 |
| Age >50 yrs. | Age <35 yrs. | 0.024 | 0.002 | -1.411 | 1.458 | 0.958 | 0.517 | 1.934 |
| Children | none | 0.346 | 0.034 | -0.649 | 1.342 | 0.509 | 0.544 | 1.837 |
| Burden of care | none | 0.095 | -0.005 | -1.382 | 1.571 | 0.295 | 0.920 | 1.087 |
| Working in a station originally piloting | no | -0.208 | -0.018 | -1.062 | 0.645 | 0.015 | 0.973 | 1.028 |
| Patrol duty | office duty | 0.265 | 0.024 | -0.615 | 1.145 | 0.789 | 0.782 | 1.280 |
| *Modell parameters* n=758, R²= 0.005, adjusted R²= -0.005, p=0.848, average VIF= 1.433 | | | | | | | | |
| B: non-standardized coefficient; Beta: standardized coefficient; LL: lower limit; UL: upper limit; VIF: variance inflation factor. | | | | | | | | |

**Supplementary Table 8. Long-term follow-up – Multivariate analysis WHOQOL-Bref Global.**

|  | **Reference** | **Coefficients** | | **95% CI for B** | | **p** | **Collinearity** | |
| --- | --- | --- | --- | --- | --- | --- | --- | --- |
|  |  | **B** | **Beta** | **LL** | **UL** |  | **Tolerance** | **VIF** |
| Constant |  | 72.267 |  | 66.263 | 78.272 | <0.001 |  |  |
| Length of work with remodelled shift (months) | (cont.) | -0.065 | -0.063 | -0.144 | 0.015 | 0.112 | 0.800 | 1.251 |
| Gender | male | -1.435 | -0.038 | -4.209 | 1.339 | 0.310 | 0.919 | 1.089 |
| Age 35 -49 yrs. | Age <35 yrs. | -1.508 | -0.041 | -5.153 | 2.136 | 0.417 | 0.510 | 1.962 |
| Age >50 yrs. | Age <35 yrs. | 0.132 | 0.003 | -4.830 | 5.094 | 0.958 | 0.515 | 1.942 |
| Children | none | -1.161 | 0.032 | -4.611 | 2.290 | 0.509 | 0.545 | 1.835 |
| Burden of care | none | -2.724 | -0.039 | -7.826 | 2.379 | 0.295 | 0.931 | 1.074 |
| Working in a station originally piloting | no | 3.869 | 0.089 | 0.729 | 6.650 | 0.015 | 0.971 | 1.030 |
| Patrol duty | office duty | 0.415 | 0.011 | -2.632 | 3.463 | 0.789 | 0.783 | 1.278 |
| *Modell parameters* n=776, R²= 0.020, adjusted R²= 0.010, p=0.044, average VIF= 1.432 | | | | | | | | |
| B: non-standardized coefficient; Beta: standardized coefficient; LL: lower limit; UL: upper limit; VIF: variance inflation factor. | | | | | | | | |

**Supplementary Table 9. Long-term follow-up – Multivariate analysis Quality of life with shift model (Score 0-10).**

|  | **Reference** | **Coefficients** | | **95% CI for B** | | **p** | **Collinearity** | |
| --- | --- | --- | --- | --- | --- | --- | --- | --- |
|  |  | **B** | **Beta** | **LL** | **UL** |  | **Tolerance** | **VIF** |
| Constant |  | 6.740 |  | 6.207 | 7.273 | <0.001 |  |  |
| Length of work with remodelled shift (months) | (cont.) | -0.003 | -0.063 | -0.010 | 0.004 | 0.398 | 0.802 | 1.246 |
| Gender | male | 0.022 | -0.006 | -0.224 | 0.268 | 0.861 | 0.919 | 1.088 |
| Age 35 -49 yrs. | Age <35 yrs. | -0.522 | -0.157 | -0.847 | -0.197 | 0.002 | 0.506 | 1.977 |
| Age >50 yrs. | Age <35 yrs. | -0.543 | -0.120 | -0.983 | -0.102 | 0.016 | 0.513 | 1.950 |
| Children | none | 0.303 | 0.093 | -0.004 | 0.610 | 0.053 | 0.542 | 1.845 |
| Burden of care | none | -0.115 | -0.018 | -0.566 | 0.335 | 0.616 | 0.928 | 1.078 |
| Working in a station originally piloting | no | 0.309 | 0.083 | 0.046 | 0.572 | 0.021 | 0.971 | 1.030 |
| Patrol duty | office duty | 0.180 | 0.052 | -0.090 | 0.450 | 0.192 | 0.783 | 1.278 |
| *Modell parameters* n=778, R²= 0.033, adjusted R²= 0.023, p=0.001, average VIF= 1.437 | | | | | | | | |
| B: non-standardized coefficient; Beta: standardized coefficient; LL: lower limit; UL: upper limit; VIF: variance inflation factor. | | | | | | | | |

**Supplementary Table 10. Long-term follow-up – Multivariate analysis Self-rated health status (Score 0-10).**

|  | **Reference** | **Coefficients** | | **95% CI for B** | | **p** | **Collinearity** | |
| --- | --- | --- | --- | --- | --- | --- | --- | --- |
|  |  | **B** | **Beta** | **LL** | **UL** |  | **Tolerance** | **VIF** |
| Constant |  | 7.306 |  | 6.785 | 7.828 | <0.001 |  |  |
| Length of work with remodelled shift (months) | (cont.) | 0.000 | -0.005 | -0.007 | 0.006 | 0.902 | 0.799 | 1.251 |
| Gender | male | -0.030 | -0.009 | -0.271 | 0.212 | 0.810 | 0.918 | 1.089 |
| Age 35 -49 yrs. | Age <35 yrs. | -0.246 | -0.076 | -0.562 | 0.071 | 0.128 | 0.510 | 1.961 |
| Age >50 yrs. | Age <35 yrs. | -0.330 | -0.075 | -0.761 | 0.101 | 0.134 | 0.515 | 1.942 |
| Children | none | -0.017 | -0.005 | -0.316 | 0.283 | 0.913 | 0.545 | 1.834 |
| Burden of care | none | -0.006 | -0.001 | -0.449 | 0.437 | 0.978 | 0.932 | 1.074 |
| Working in a station originally piloting | no | 0.177 | 0.049 | -0.080 | 0.434 | 0.177 | 0.971 | 1.030 |
| Patrol duty | office duty | 0.197 | 0.059 | -0.068 | 0.462 | 0.145 | 0.783 | 1.277 |
| *Modell parameters* n=775, R²= 0.019, adjusted R²= 0.009, p=0.061, average VIF= 1.432 | | | | | | | | |
| B: non-standardized coefficient; Beta: standardized coefficient; LL: lower limit; UL: upper limit; VIF: variance inflation factor. | | | | | | | | |

**Supplementary Table 11. Scores according to attitude regarding change to the remodelled shift schedule.**

|  | **T0 (05/2015)** | | | | | **T1 (06/2016)** | | | | | | **Change (T1-T0)** | | | | |
| --- | --- | --- | --- | --- | --- | --- | --- | --- | --- | --- | --- | --- | --- | --- | --- | --- |
|  | **against** | | **for** | |  | **against** | | **for** | | |  | **against** | | **for** | |  |
|  | **mean** | **SD** | **mean** | **SD** | **p** | **mean** | **SD** | **mean** | **SD** | **p** | | **mean** | **SD** | **mean** | **SD** | **p** |
| Work Ability Index (n=360) [Score 7-49] | 39.94 | 5.12 | 38.43 | 5.52 | 0.005 | 39.50 | 5.51 | 39.03 | 5.32 | 0.380 | | -0.31 | 4.01 | 0.65 | 4.92 | 0.019 |
| WHOQOL-Bref Global (n=578) [Score 0-100] | 67.67 | 18.31 | 58.76 | 18.49 | <0.01 | 66.98 | 20.22 | 64.26 | 18.87 | 0.105 | | -0.60 | 20.37 | 5.49 | 20.45 | 0.001 |
| Quality of life with shift schedule (n=567) [Score 0-10] | 6.60 | 1.69 | 4.71 | 1.72 | <0.01 | 6.25 | 1.82 | 5.65 | 2.12 | <0.01 | | -0.32 | 1.76 | 0.93 | 2.22 | <0.01 |
| Self-rated health status score (n=576) [Score 0-10] | 7.19 | 1.64 | 6.68 | 1.82 | 0.001 | 7.24 | 1.67 | 6.90 | 1.68 | 0.020 | | 0.04 | 1.46 | 0.22 | 1.82 | 0.187 |
